# Nanoparticle Uptake and Crossing by Human In Vitro Models of Intestinal Barriers: A Scoping Review

**DOI:** 10.3390/nano15151195

**Published:** 2025-08-05

**Authors:** Chiara Ritarossi, Valentina Prota, Francesca De Battistis, Chiara Laura Battistelli, Isabella De Angelis, Cristina Andreoli, Olimpia Vincentini

**Affiliations:** 1 Environment and Health Department, Istituto Superiore di Sanità, 00161 Rome, Italy; chiara.ritarossi@iss.it (C.R.); chiara.battistelli@iss.it (C.L.B.); cristina.andreoli@iss.it (C.A.); 2 Food Safety, Nutrition and Veterinary Public Health Department, Istituto Superiore di Sanità, 00161 Rome, Italy; valentina.prota@iss.it (V.P.); francesca.debattistis@iss.it (F.D.B.)

**Keywords:** in vitro intestinal barrier, human exposure, crossing, uptake, nanoparticles, new approach methodologies

## Abstract

The Caco-2 in vitro model of the intestinal barrier is a well-established system for the investigation of the intestinal fate of orally ingested chemicals and drugs, and it has been used for over ten years by pharmaceutical industries as a model for absorption in preclinical studies. The Caco-2 model shows a fair correlation with in vivo drug absorption, though some inherent biases remain unresolved. Its main limitation lies in the lack of structural complexity, as it does not replicate the diverse cell types and mucus layer present in the human intestinal epithelium. Consequently, the development of advanced in vitro models of the intestinal barrier, that more structurally resemble the human intestinal epithelium physiology, has increased the potential applications of these models. Recently, Caco-2-based advanced intestinal models have proven effective in predicting nanomaterial uptake and transport across the intestinal barrier. The aim of this review is to provide a state-of-the-art of human in vitro intestinal barrier models for the study of translocation/uptake of nanoparticles relevant for oral exposure, including inorganic nanomaterials, micro/nano plastic, and fiber nanomaterials. The main effects of the above-mentioned nanomaterials on the intestinal barrier are also reported.

## 1. Introduction

The growing use of nanomaterials (NMs) across sectors such as healthcare, pharmaceutical, and the food industry has raised concerns about their potential risks to human health and the environment. Traditional risk assessment methods, largely reliant on animal testing, face limitations when applied to NMs due to their unique physicochemical properties and nanoscale behaviors. Since the onset of NM research, considerable emphasis has been placed on evaluating their safety across different areas of applications.

Although a unified international definition of NMs is still lacking, regulatory bodies, such as the International Organization for Standardization (ISO), the OECD Working Party on Manufactured Nanomaterials (OECD-WPMN), and the European Commission, generally define an NM as a material with external dimensions, internal structure, or surface structure in the 1–100 nm range.

The terms nanoparticle (NP) and NM are related but not interchangeable. While the term ‘nanoparticle’ refers specifically to materials with all three external dimensions in the nanoscale, ‘nanomaterial’ is a broader regulatory term encompassing particles, unbound or in aggregates/agglomerates, of which ≥50% fall within the nanoscale. This includes NPs, nanofibers, and other nanoforms. Regulatory frameworks therefore prefer the term ‘NMs’ to ensure comprehensive coverage for safety and risk assessment [1].

Nanoscale dimensions significantly affect material behavior, increasing surface area and reactivity as the particle size decreases, which can enhance toxicity to the respective non-nanoform. Smaller NMs more easily penetrate tissues and cells, and even well-characterized substances may exhibit unusual physicochemical and biological properties at this scale [2]. It is necessary to properly assess and manage any potential risk linked to the nano-specific properties.

In recent years, New Approach Methodologies (NAMs) are gaining increasing acceptance in the hazard assessment of chemicals, pharmaceuticals, and other consumer products. NAMs include a wide range of approaches such as in vitro models, in silico methods, omics technologies, high-throughput screening (HTS) assays, and organ-on-a-chip applications. Many of the techniques categorized as NAMs have existed for decades, but only in recent years they have been integrated into risk assessment procedures; therefore, the novelty is their application into regulatory contexts [3].

Although the definition of NAMs may differ slightly depending on the context and the organization using it, there is broad consensus that NAMs are designed to reduce or replace animal testing, while enhancing the efficiency, reliability, and human biological relevance of toxicological assessments [4,5]. These innovative approaches play a key role in NM risk assessment; for instance, in the European Food Safety Authority (EFSA) Guidance on risk assessment of NMs in the food and feed sector, NAMs are proposed as the first choice to generate the required information and improve mechanistic understanding of processes at the nanoscale [6]. Oral ingestion is one of the most relevant exposure routes for NMs, as they are commonly incorporated into food additives and packaging to improve product quality, safety, and stability [7].

Biological barriers play a crucial role in protecting the human body from toxicants by limiting or preventing the entry of harmful substances and pathogens into systemic circulation. Key barriers include the skin, respiratory tract, gastrointestinal tract (GIT), blood–brain barrier, and placenta. Advanced in vitro models of these barriers, such as co-cultures, organoids, and reconstructed tissues, have emerged as an essential component of NAMs in toxicology and safety testing. One of the most promising areas where NAMs have demonstrated substantial potential is in the development of advanced in vitro models that closely replicate the human intestinal barrier. These models enable a more realistic simulation of NM exposure and provide valuable insights into their ability to cross the intestinal barrier, which is critical for understanding both acute and chronic toxicological effects [7]. Human intestinal cell lines are widely used in these models for pharmacological and toxicological applications as well as for uptake and translocation studies for both chemicals and NMs. Caco-2 cells are a well-established model of human enterocytes, widely used for determining active and passive absorption of drugs and chemicals.

Caco-2 cells, grown on permeable inserts, can spontaneously differentiate in long term culture in enterocytes, i.e., displaying microvilli, tight junctions (TJ), hydrolyzing enzymes, and carrier-mediated transport systems responsible for nutrient absorptions [8]. They are generally considered the best starting point for in vitro models of the intestinal barrier. The Caco-2 model is recognized by several international regulatory organizations, including the OECD, the European Medicines Agency (EMA), and the Food and Drug Administration (FDA), as a suitable intestinal barrier model for chemical permeability studies [9,10,11].

However, the Caco-2 monoculture model shows several limitations, mainly due to the greater structural complexity of the intestinal epithelium. As a result, the development of co-culture models with varying levels of complexity, combining different intestinal cell types, has significantly enhanced model’s applicability [12,13]. In particular, the simultaneous presence of mucus-secretory cells (HT29-MTX), that can have an impact on NMs mobility, and of hematopoietic cells (Raji-B cells), able to promote Caco-2 conversion in specialized M cells involved in particulate uptake, or immune-competent cells (differentiated THP-1 cells), can enhance the physiological relevance of the model.

This review is focused on the interaction of different categories of NMs, all relevant for oral exposure, providing an overview of the existing literature data on NMs’ uptake, crossing, and impairment of the intestinal barrier integrity mainly on Caco-2-based barrier models. An overview of the results on the above parameters for each NM is provided. Both inorganic (metallic and non-metallic) and organic polymeric NMs including nanocelluloses (NCs) and micro and nanoplastics (MNPs) are examined. Moreover, studies focused on the effects of NMs subjected to in vitro-simulated digestion have been also considered. NMs’ in vitro-simulated digestion process involves sequential exposure to artificial salivary, gastric, and intestinal fluids, aiming to replicate the conditions of the human GIT and to evaluate the stability, biotransformation, and potential bioavailability of NMs following the digestive process. This simulated digestion allows us to obtain more detailed information on NMs’ intestinal behavior in physiological conditions closer to reality.

## 2. Methods

For this scoping review, a comprehensive literature search was performed to identify all the studies related to the impact of food-relevant NMs on intestinal barriers and their potential crossing and uptake. It was designed using the following steps:

- Developing a search strategy
- Determining the inclusion and exclusion criteria
- Identifying the relevant studies through database search
- Screening and paper selection
- Extraction of the data
- Summarizing and reporting the results

### 2.1. Search Strategy

An overview of the available literature for testing NMs uptake and translocation across the intestinal barrier was performed using published scientific literature resources on PubMed and Scopus databases. Regulatory documents from international organizations and agencies available from 2015 to 2025 have been served as supporting documents [6,11,14]. According to criteria and templates previously defined, state-of-the-art analysis has been carried out using published scientific literature resources focusing on NMs relevant for oral exposure in food sector. Intestinal barrier integrity, uptake, and the translocation of both inorganic NPs, such as titanium dioxide, zinc oxide, silicon dioxide, silver, aluminum, cerium dioxide, iron, copper oxide, gold, and organic NPs, such as micro- and nanoplastics and nanofibers, have been considered.

The first search was performed using the general term “nanoparticle” related to barrier integrity, uptake/accumulation, and crossing/translocation, and then a specific search was carried out for each type of material analyzed. In this first search there were 551 original papers found. The number of papers retrieved for each “search terms” is reported in Table 1. More detailed information on the number of papers retrieved through the search strategy is provided in Table S1 (Supplementary Materials).

Once the papers have been identified, before the “screening phase”, an initial skimming of the papers was completed. Non-English or duplicate papers were discarded, leaving 263 articles to be submitted to the next “screening phase”.

In order to assess the paper’s suitability for the purposes of this review, appropriate inclusion/exclusion criteria were adopted at this “screening phase”.

Inclusion criteria consider elements related to the type of experimental model; for example, only human-related studies or in vitro studies were included. Moreover, elements related to the focus of the reported study, such as studies investigating uptake or transport processes, were considered. Additionally, the source and description of the NPs used in the studies were included. Lastly, the analysis considered only peer-reviewed papers. Exclusion criteria cover the same categories, considering for example, not human-focused model types; regarding the focus of the study, papers not evaluating NP uptake or crossing were excluded; moreover, studies focused on non-nanoforms were excluded; lastly, regarding the publication type, non-original research, grey literature, and review papers were discarded. The papers excluded, according to the inclusion/exclusion criteria, were 192. An exhaustive list of elements considered as inclusion and exclusion criteria is reported in Table 2.

**Figure 1:**
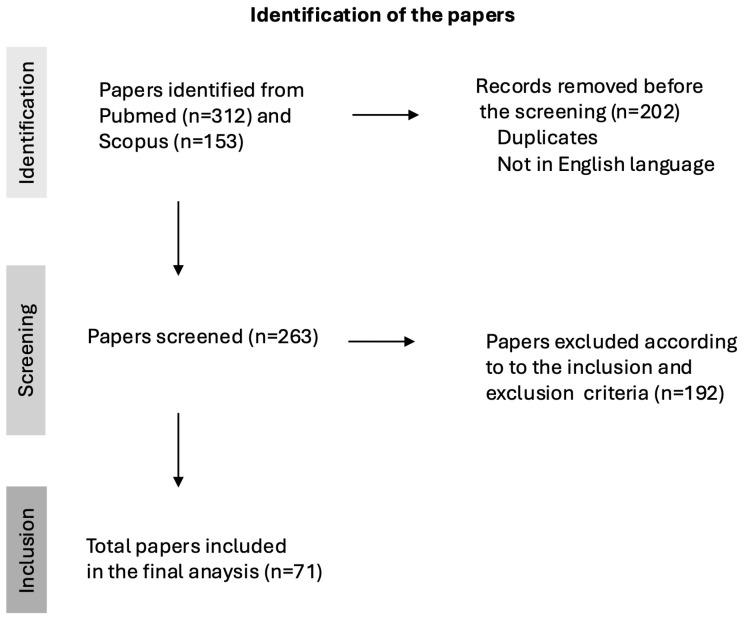
Scoping review procedure for identifying papers: sequence of steps and number of articles included/excluded in each step. Modified from the Preferred Reporting Items for Systematic reviews and Meta-Analyses (PRISMA) statement [15].

In total, 71 papers were submitted for the final analysis. Figure 1 depicts the flow chart of this scoping review, showing the sequence of steps, i.e., identification, screening and inclusion of papers, and the number of articles included or excluded in each step.

### 2.2. Parameters and Endpoints Considered for Barriers Models

The main parameters and endpoints taken into consideration for this review are listed in Table 3. They are divided into the following three main groups: NMs’ physical–chemical parameters, experimental parameters, and endpoints.

## 3. Results

This section summarizes the relevant results reported in the analyzed papers in terms of uptake, crossing, and impairment of the intestinal barrier for both inorganic and organic NMs. All the reviewed studies employed human intestinal epithelial cell lines, such as Caco-2 cells or co-culture models grown on permeable membrane inserts (Transwell^®^ systems). In addition to pristine NMs, studies that investigated the effects of NMs subjected to simulated gastrointestinal digestion were also evaluated.

Variability in experimental designs, cell types, and NM exposure conditions contributes to the heterogeneity of the observed effects. Table S2 (Supplementary Materials) reported the key details of the studies examined, including authors, publication year, types and size of NMs, types and characterization of the in vitro barrier models including cell lines provider, NM treatment dose and time of exposure, uptake, and crossing results.

### 3.1. Inorganic Nanomaterial

Given the increasing dietary exposure to inorganic NMs, in vitro intestinal barrier models have become essential tools for evaluating the interactions of inorganic NPs with the gastrointestinal epithelium providing a controlled environment to study NP uptake, translocation, cytotoxicity, and effects on barrier integrity.

Notwithstanding, studies on inorganic NPs tested on in vitro intestinal barrier models are quite limited. This review compiles and analyses findings from studies on inorganic NPs, as titanium dioxide, zinc oxide, silicon dioxide, silver, aluminum, cerium dioxide, iron, copper oxide, and gold, in various shapes and sizes (<250 nm), as presented in the following sections.

#### 3.1.1. Titanium Dioxide

Titanium dioxide (TiO_2_—food additive E171) has been used as a food additive in the EU for over 50 years, mainly in products like pastries and sweets for its whitening properties. Composed mostly of anatase particles (some up to 250 nm), TiO_2_ is biopersistent and resists lysosomal degradation. While inhalation effects are well-studied, oral exposure is less understood, though particles can accumulate in the body over time despite low absorption [16]. E171 can enter the bloodstream after ingestion and accumulate in organs due to its long biological half-life. Despite low gut absorption, its nanoscale properties contribute to potential health risks, including inflammation, immune disruption, neurological effects, DNA damage, and genotoxicity. Based on new scientific evidence, the European Food Safety Authority (EFSA) has reassessed its safety and concluded that E171 can no longer be considered safe for human consumption [17]. The decision is based on new scientific evidence indicating the potential genotoxicity of ingested TiO_2_ particles. Although gut absorption is low, the particles can accumulate in the body over time, raising concerns about possible long-term health effects.

These concerns ultimately led to its ban as a food additive in the EU in 2022 [18], but it remains permitted in other products like toothpaste and sunscreen.

Only recently studies have begun to shed light on the uptake and translocation of ingested TiO_2_ across the GIT [19]. After ingestion, NPs can be absorbed through various mechanisms, with endocytosis-related pathways at the mucosal membrane being the most widely recognised for the uptake of poorly soluble nano-TiO_2_. Previous papers demonstrated whether factors such as crystal structure and particle size affect how gut cells absorb TiO_2_ in in vitro intestinal models. Moreover, it was demonstrated that uptake in simple models (Caco-2 monoculture) was very low. Conversely, accumulation was found in more complex models such as the goblet model (Caco-2/HT-29 MTX) and the co-culture Caco-2/Raji B cells [20,21].

The impact of NP exposure (250 μg/mL for 24 h) on two in vitro models of gut epithelium was assessed, as follows: a monoculture of Caco-2 cells and a co-culture of Caco-2 and RajiB cells to mimic M-cells [22]. To assess the potential translocation of TiO_2_ NPs (<100 nm), both apical (AP) and basolateral (BL) compartments were analyzed using single-particle inductively coupled plasma mass spectrometry (sp-ICP-MS). They found no evidence of TiO_2_ translocating across the intestinal epithelial barrier. The TiO_2_ particles appeared to accumulate either between or within the cells, as TiO_2_ NPs were detected in the cellular fractions but not in the BL compartment. Furthermore, the exposure did not critically affect the integrity of the monolayer. The same research group reported that in vivo oral administration of TiO_2_, whether in nano or larger particle form, did not lead to significant internal exposure in rats, suggesting the minimal absorption of the particles through dietary intake.

The biointeractions, biodistribution, and toxicokinetics of three differently shaped TiO_2_ NPs—nanospheres (anatase <25 nm—TiO_2_ NPs-S), nanorods (rutile <100 nm diameter, ~250 nm length- TiO_2_ NPs-R), and nanowires <10 nm diameter, ~100 μm length—TiO_2_ NPs-W)—were studied in an in vitro Caco-2/HT29 intestinal barrier model [23]. Exposure was conducted at 12.5, 50, 100, and 350 μg/mL for 24 and 48 h. Barrier integrity, paracellular permeability, and cellular uptake and localization by confocal microscopy were analyzed. TiO_2_ NPs-W and TiO_2_ NPs-R significantly disrupted barrier integrity, particularly at 150 μg/mL, while TiO_2_ NPs-S had negligible effects at 24 h exposure. All NPs increased paracellular transport, with TiO_2_ NPs-W being the most disruptive across multiple conditions. After 24 h exposure, most of the TiO_2_ NPs remained on the AP side. TiO_2_ NPs-S uptake increased over time, while TiO_2_ NPs-R showed time- and concentration-dependent uptake. TiO_2_ NPs-W showed unique behavior, and transport increased over time but decreased at higher concentrations. Confocal imaging showed NPs localized in AP mucus, cytoplasm, and occasionally in nuclei. Crossing of the barrier was detected for all three NPs, indicating the ability of TiO_2_ NPs to pass through the in vitro intestinal barrier, independently of their shape. Both TiO_2_ nanospheres and nanorods were taken up easier and faster than nanowires, using transcellular transport to cross the barrier model.

In another study, the effects of TiO_2_ NPs (Joint Research Center, JRC, repository NM-100) on both undifferentiated and differentiated Caco-2 cells across a concentration range of 1–200 μg/mL were investigated [24]. Authors found no cytotoxicity at any tested concentration and no impact on barrier integrity. Uptake and translocation were observed in differentiated monolayers using confocal and electron microscopy coupled with Energy Dispersive X-Ray Spectroscopy (EDX) with a higher NPs uptake in undifferentiated cells probably, since the thickness of undifferentiated cells is much less than differentiated cells. In differentiated Caco-2 monolayers, TiO_2_ NPs largely remained apically bound, likely retained in microvilli and the extracellular matrix, and showed limited internalization. Although confocal microscopy and Transmission Electron Microscopy (TEM) coupled with EDX detected some TiO_2_ NP translocation across the monolayer and the insert membrane, the amount of titanium in the BL chamber was below the ICP-MS detection limit.

Some attempts have been made with spheroids from Caco-2 cells [25]. Caco-2 spheroids were repeatedly treated with TiO_2_ NPs at concentrations from 15.7 ng/mL to 1.57 μg/mL to simulate chronic exposure. Treatments occurred on days 3, 5, and 7, and spheroids were exposed until day 9. A dose-dependent increase in the concentration of Ti inside 3D Caco-2 spheroids after repeated exposure has been found; although, this could not be distinguished by ICP-MS internalized NPs and NPs adhered to a cellular membrane.

More recently, a study investigated mechanisms associated with uptake, epithelial barrier function, intracellular movement, and activation of inflammatory pathways following exposure to three TiO_2_ NPs from the JRC repository: NM-102 (anatase 400 nm), NM-103 (rutile 120 nm), and NM-105 (mixture of anatase and rutile 130 nm), both in Caco-2 and HT-29 MTX cells [26]. The experiments demonstrated that all three NPs, whether digested or not, were clearly visible within the cytoplasm of HT29-MTX-E12 cells, whereas only occasional signals of internalized NPs were observed in Caco-2 cells. At a physiologically relevant concentration of 14 μg/mL, the digested TiO_2_ NPs did not impair transepithelial electrical resistance (TEER) or affect the expression of key epithelial markers such as E-cadherin and Zonula Occludens-1 (ZO-1) in polarized enterocyte monolayers. Despite this, all NPs were taken up by the cells. NM-102 accumulated within lysosomes, whereas NM-103 and NM-105 were able to cross the epithelial barrier via transcytosis. Furthermore, a 24 h exposure to digested NM-105 at concentrations of 14 and 1.4 μg/mL triggered IL-1β production in activated M1 macrophages, suggesting a potential pro-inflammatory effect in the gut. No passage of TiO_2_ NPs was detected at low doses but only at higher, supra-physiological concentrations (100 µg/mL).

In conclusion, EFSA declared E171 (food-grade TiO_2_) unsafe due to concerns over genotoxicity and accumulation in the body [17], leading to its ban in the EU [18]. TiO_2_ particles, especially at nanoscale (200–300 nm), can enter the bloodstream and accumulate in organs. Adverse effects include inflammation, immune disruption, neurological effects, DNA damage, and intestinal abnormalities (e.g., aberrant crypt foci). Oral absorption is low, but accumulation is possible, and endocytosis is a key uptake route, especially for nano-TiO_2_. Physicochemical properties (e.g., size, shape, crystal structure) have been shown to affect absorption and translocation. In simple models as in Caco-2 monoculture, low TiO_2_ uptake and minimal translocation have been detected. In fact, negligible transport though some AP binding and limited internalization were observed [22,24]. Conversely complex models such as Caco-2/HT29-MTX (mucus-producing) and Caco-2/Raji B (M-cell) models showed greater accumulation and occasional translocation. In detail, authors found shape-dependent uptake with nanowires (TiO_2_ NPs-W) causing the most barrier disruption [23]. Overall, all NP types crossed the epithelial barrier, mainly via transcellular transport.

#### 3.1.2. Zinc Oxide

Zinc oxide (ZnO) is an essential mineral for the body and due to its characteristics as antimicrobial and photocatalytic activity and mechanical, thermal, and chemical stability, it is widely used in the food sector as a food additive acting as a zinc (Zn) fortifier, a dietary supplement, and a food contact material.

It is almost insoluble in water, but it can undergo dissolution under the acidic pH conditions characteristic of the human digestive system. This dissolution may affect the toxicological profile and the biological fate of the ZnO NPs at the intestinal level. It is already known that zinc is predominantly absorbed in the small intestine, but there is scarce knowledge about the uptake and fate of ZnO NPs in the human GIT [27]. To evaluate the intestinal uptake of ZnO NPs and its possible effects on the GIT, in vitro-simulated digestion process is a relevant aspect to be considered for the evaluation of the fate of ZnO after ingestion. Available data on the possible toxicity of ZnO at the intestinal level are heterogeneous [28], and they seem to be related to the cell culture model used, among other factors [29]. The safety of food products containing ZnO NPs in food packaging has been investigated by extracting ZnO from canned food and evaluating its effect on a human in vitro intestinal model composed of Caco-2/HT29-MTX co-culture grown for 2 weeks on 0.4 µm pore size Transwell^®^ system [30]. Cells were exposed for 4 h to both pristine and digested ZnO NPs to reflect the transformation that NPs undergo in the GIT. The authors observed that ZnO NPs dissolve in cell culture medium or during the in vitro digestion process. ZnO NPs’ effects on paracellular permeability and cellular viability were assessed. A significant increase in the TEER of all concentrations (1 × 10^9^, 1 × 10^11^ and 1 × 10^13^ NP/cm^2^) of digested NPs compared to the undigested NPs was observed. In cells exposed to digested ZnO NPs, a slight increase in the intestinal alkaline phosphatase (IAP) was also observed, whereas a decrease in IAP was noted following exposure to undigested ZnO NPs. Scanning electron microscopy (SEM) revealed the disruption of the brush border membrane at higher concentrations of digested ZnO NPs.

The fate and the effects of two different sized (<50 nm and <100 nm) in vitro digested ZnO NPs were investigated on Caco-2 monoculture and Caco-2/HT29-MTX co-culture [28]. Cellular in vitro models were grown for 23 days on 3 μm pore size insert and were then exposed to in vitro-digested ZnO ranging from 123 to 614 µM. After in vitro digestion, a chemical transformation with about 70% free zinc ions was reported using Inductively Coupled Plasma Optical Emission Spectroscopy (ICP-OES). Cellular uptake of digested ZnO NPs and zinc permeation across the cellular monolayer were quantified using ICP-MS, revealing a dose-dependent increase in intracellular zinc levels—up to a threefold increase in the monoculture and a fourfold increase in the co-culture model. Indeed, a low amount of zinc was detected in the BL compartment, indicating that the integrity of the cellular monolayer was maintained, limiting uncontrolled zinc translocation across the barrier. No changes in TEER values were observed in Caco-2 monocultured and Caco-2/HT29-MTX co-culture after 24 h treatment with digested ZnO NPs. Moreover, digested ZnO NPs were reported to have no impact on paracellular permeability measured by Fluorescein isothiocyanate-labelled dextran (FITC-dextran) administered apically. No influence of ZnO NP on the cytoskeletal and nuclear morphology was also observed.

Moreover, it was analyzed how the interactions between ZnO NPs (Sigma-Aldrich, Darmstadt, Germany; particle size 78 ± 26 nm) and food proteins, such as albumin from chicken egg white, casein from bovine milk, and zein from corn, can modulate in vitro biological responses [31]. To assess intestinal transport, a Caco-2 monoculture model and a follicle-associated epithelial (FAE) model mimicking microfold (M) cells by Caco-2/Raji B co-culture were used. On day 19 of co-culture, a 6 h treatment with ZnO NPs (50 µg/mL) was performed. The interaction between ZnO NPs and albumin leads to a reduction in NP hydrodynamic diameter, as measured by dynamic light scattering (DLS), which was associated with enhanced intestinal transport. In contrast, interactions with casein or zein led to the formation of aggregates, without significantly affecting the extent of in vitro intestinal transport. The intestinal transport of ZnO NPs was always higher in the Caco-2/Raji B co-culture than in Caco-2 monoculture.

In another study, Caco-2 monoculture and a co-culture of Caco-2/HT29-MTX were exposed to ZnO NPs (<50 nm and <100 nm) at concentrations of 123–614 µmol/L, after 21 days of culture on 3 µm pore size inserts [32]. Barrier integrity was evaluated by TEER measurements. In Caco-2 monocultures, ZnO NP treatment did not affect TEER values. Conversely, in co-culture models, TEER values increased significantly following exposure to ZnO NPs at concentrations ≥307 µmol/L. No significant increase in transepithelial permeability was observed after 24 h of ZnO NP exposure, indicating the preservation of intestinal barrier integrity. Furthermore, phalloidin and DAPI staining revealed no notable alterations in cytoskeletal organization or nuclear morphology following treatment. Zinc uptake was higher in Caco-2 monocultures compared to Caco-2/HT29-MTX co-cultures. Only a small amount of zinc (0.07–0.65 µg/mL) was detected in the BL compartment after 24 h, suggesting limited translocation of ZnO NPs across the intestinal barrier.

Furthermore, the toxicological effects of several food-grade NPs, including ZnO NPs, were investigated using Caco-2 monoculture, Caco-2/HT29-MTX co-culture, and Caco-2/HT29-MTX/Raji B triculture models cultured on 0.4 µm pore-size inserts, at a dose of 4.05 µg/cm^2^ [29]. No significant changes in TEER values were observed in the triculture model following NP exposure, whereas monocultures exhibited up to a 50% reduction in TEER, indicating compromised barrier integrity. Instead, decreased cell viability and metabolic activity were higher at lower doses in the co-culture and tri-culture model compared to the monoculture. Moreover, a significant increase in pro-inflammatory cytokine production, including IL-6, IL-1β, and IL-8, was observed in co- and tri-culture models compared to monoculture. Among the food-grade NPs tested, ZnO was found to be the most cytotoxic.

The interaction between ZnO NPs and additive solvents as methanol, glycerin, and propylene glycol, has been investigated in term of ZnO NP intestinal transport on two different in vitro intestinal epithelium models grown on a 3 µm pore size insert, Caco-2 monolayer and Caco-2/Raji B co-culture [33]. Both intestinal models were exposed to 50 µg/mL of ZnO NPs (<100 nm) in 1% additive solvent for 6 h. A significant increase in the intestinal transport of ZnO NPs was observed in both monoculture and co-culture models in the presence of glycerin or propylene glycol, to a greater extent in the co-culture. The hydrodynamic diameter of ZnO NPs decreased, and their solubility increased upon interaction with glycerin and propylene glycol, whereas no such effects were observed with methanol.

The apparent permeability coefficient (Papp) and TJ integrity were measured on Caco-2 monoculture grown for 16 days on 3 μm pore size insert after 24 h exposure to 50 μg/mL ZnO NPs [34]. A significative increase in Papp was observed after 24 h exposure to ZnO NPs, while no major reorganization of ZO-1 was reported by immunofluorescence microscopy. No cytotoxicity in terms of cell membrane integrity and mitochondrial and metabolic activity were reported after 24 h treatment with ZnO NPs.

In conclusion, based on the analyzed studies, ZnO NPs appear to be taken up by intestinal epithelial cells in in vitro models, but their uptake and effects depend on multiple factors, such as particle size, digestion process, and the complexity of the cell culture models. The uptake does not necessarily result in adverse effects on barrier function or cell viability, particularly in co-culture models compared to monoculture. The intact epithelial barrier appears to limit the translocation of zinc to the BL side, suggesting that the intestinal epithelium can act as a protective barrier against NP translocation under certain conditions.

#### 3.1.3. Silicon Dioxide

Silicon dioxide (SiO_2_) NPs are nanoscale materials composed of silicon dioxide, commonly found in quartz and sand. Due to their small size, large surface area, and high reactivity, they have diverse applications in food and feed production (food additive E551), packaging, personal care, pharmaceuticals, drug delivery, biomedical imaging, and electronics. Following oral exposure, the gastrointestinal absorption of SiO_2_ NPs is generally low and influenced by particle size, surface properties, and aggregation state. Most SiO_2_ NPs remain in the gut lumen, with systemic absorption occurring at low levels following intestinal barrier translocation. Once absorbed, they may accumulate in organs such as the liver, spleen, and kidneys. Therefore, a thorough toxicity assessment is crucial to ensuring their safe use [35,36].

A study evaluated the uptake of 24.7 nm SiO_2_ NPs (150 μg/mL; JRC repository NM-203) after 24 h of exposure using a Caco-2/HT29-MTX/Raji B tri-culture intestinal model. Laser scanning confocal microscopy revealed that the NPs penetrated the mucus layer, crossed the cell membrane, and ultimately reached the cell nucleus [23]. In four of the reviewed studies, SiO_2_ NPs larger than 50 nm did not compromise intestinal barrier integrity, as assessed by TEER and/or the paracellular transport of markers, such as Lucifer Yellow (LY) and FITC-dextran [37,38,39,40]. One study exposed, for 6 and 9 h, Caco-2 monocultures to 100 μg/mL of fluorescently labeled SiO_2_ NPs (50 and 150 nm), characterized by differential centrifugal sedimentation (DCS). No significant changes in TEER were observed. Uptake studies using flow cytometry and TEM revealed limited internalization in both undifferentiated (4-day culture) and differentiated (21-day culture) Caco-2 cells, with higher NPs association in the undifferentiated cells. This suggests that cell differentiation reduces NPs uptake over time. Translocation across the barrier was negligible in both models [39]. Strugari and collaborators evaluated the effects of 250–300 nm silicon quantum dots (20 μg/mL), characterized by DLS and TEM, on the Caco-2/HT29 co-culture model. After 3 h of exposure, no impairment in barrier integrity was detected via TEER and LY assays [38]. Additionally, Gautam et al. used a full-thickness 3D human intestinal model (EpiIntestinal™) to assess repeated exposure to 80 nm SiO_2_ NPs (25–100 μg/mL) over 72 h. Characterization was performed using TEM, energy-dispersive X-ray spectroscopy (EDS), and DLS [40]. Despite evidence of NP internalization, barrier integrity remained unaffected, as determined by TEER and FITC-dextran assays [40]. Additionally, a study investigated the effects of 160 nm, synthesized smaller rod-like mesoporous SiO_2_ NPs (srNPs) at a concentration of 600 μg/mL for 6 h on Caco-2/HT29-MTX co-culture model. The results demonstrated cellular uptake of the srNPs, both in the presence and absence of mucus, as assessed by Lionheart FX automated microscopy, without any detectable impairment of barrier integrity, as evaluated by TEER and LY assays [37]. Two studies compared the effects of SiO_2_ NPs exposure on different in vitro intestinal barrier models, evaluating NP uptake in each case [7,41]. In the study by Cornu et al., the oral toxicity of the food additive E551 (268 nm) and pristine and fluorescent SiO_2_ NPs (NP10, NP30, NP70, NP200; sizes ranging from 10 to 200 nm, characterized by DLS) was assessed after 2 h of exposure at concentrations between 1 and 10 mg/mL on Caco-2 monoculture and Caco-2/HT29-MTX co-culture models. NP10 and NP30 significantly decreased TEER and increased LY paracellular transport in both models. However, the impact on TEER was significantly attenuated in the co-culture model, likely due to the protective effect of the mucus layer. These results correlated with uptake data, showing greater internalization of NP30 in Caco-2 monoculture (71%) compared to co-culture (27%). Moreover, the TEER decrease induced by NP30 was reversible, with full recovery observed at 22 h post-treatment. No significant effects were observed for NP70, NP200, or E551. In the study by Vincentini and collaborators, fluorescent SiO_2_ NPs (50 nm, 100 μg/mL), characterized by DLS, were tested for 24 h on the following four models: Caco-2 monoculture, Caco-2/HT29-MTX co-culture, Caco-2/Raji B co-culture (M-cell model), and Caco-2/HT29-MTX/Raji B triculture. The highest translocation rate was observed in the Caco-2/Raji B model, followed by the triculture model, supporting the role of M cells as key mediators of NP translocation. Confocal microscopy also revealed that the mucus layer effectively entrapped the NPs, limiting their passage [7]. In another study, SiO_2_ NPs (NP20, NP30, NP70, NP200; 1 mg/mL) and E551 (232 nm), characterized by DLS, were incubated for 7 days with a Caco-2/HT29-MTX co-culture model [42]. E551 exposure did not affect cell viability or barrier integrity (measured by TEER). However, SiO_2_ NPs induced size-dependent effects, with NP20 and NP30 significantly reducing both parameters. Confocal microscopy showed that smaller NPs (NP20 and NP30) more readily penetrated the mucus layer, were internalized by cells, and caused cytotoxicity and increased paracellular permeability. In contrast, larger NPs (NP70 and NP200) mostly accumulated within the mucus without crossing into the epithelial layer. The results of Cornu’s and Zaiter’s papers agree with others concerning the E551 [43]. In a recent study, six food-grade SAS materials, with primary particle sizes ranging from 3 to 41 nm and aggregate sizes (TEM) from 45 to 276 nm, were tested on a Caco-2/HT29/Raji B tri-culture model. After 48 h of exposure to 50 µg/mL, none of the SAS samples caused a reduction in barrier integrity or mucus coverage, as confirmed by TEER and Alcian blue staining, respectively.

The effects of 1 mg/mL E551, subjected to in vitro-simulated digestion, were evaluated on a Caco-2/HT29-MTX co-culture after 7 days of exposure [42]. DLS analysis showed particle sizes of 232 nm for pristine E551 and 166 nm and 215 nm for the two digested batches. In line with previous findings on the pristine form, digested E551 did not induce significant toxicity in the co-culture model, indicating that simulated digestion did not alter its safety profile.

Taken together, these results suggest that smaller SiO_2_ NPs have major effects on translocation rate and/or barrier integrity. Moreover, the in vitro intestinal barrier models used results suitable for the study of SiO_2_ NPs’ potential toxic effects on human health.

In most of the analyzed papers, the treatment with SiO_2_ NPs > 50 nm did not induce barrier impairment (TEER/LY/FITC-dextran), which may be attributed to their relatively large particle size [38,39,40,43]. Most of the analyzed studies, using different in vitro intestinal barrier models (Caco-2 monoculture and Caco-2 based co-culture), confirmed the ability of SiO_2_ NPs to be taken up by cells in a size-dependent manner [7,38,40,41,42,44], and only in one paper did the authors report a weak internalization in the Caco-2 monoculture model [39]. Different in vitro intestinal barrier models exhibit varying rates of SiO_2_ NPs uptake and/or translocation. The highest translocation was observed in the Caco-2/Raji B co-culture, followed by the tri-culture model, suggesting that M-cells may serve as portals for NP translocation [7]. The mucus layer in the Caco-2/HT29-MTX model markedly reduced the uptake of SiO_2_ NPs, likely due to its barrier properties [41,42]. Finally, all studies investigating the effects of the food additive E551 consistently report no significant toxicity to intestinal barrier integrity, suggesting its safety [41,42,43], as also supported by a recent EFSA re-evaluation, which concluded that E551 does not pose a safety concern at current exposure levels, based on the available data [45].

#### 3.1.4. Silver

Silver (Ag) NPs, due to their antimicrobial properties, are among the most widely used NPs in food-related products (e.g., cooking utensils and food packaging). Consequently, oral ingestion is considered the main route of human exposure to Ag NPs [46,47,48,49]. A comprehensive risk assessment is essential to evaluate the health impact of Ag NPs, given that upon oral ingestion, these particles pass through various compartments of the GIT each characterized by distinct pH levels and biochemical compositions. These conditions may influence the physicochemical properties of Ag NPs and affect their bioavailability, cellular uptake, and toxicological properties.

To investigate the intestinal fate of Ag NPs after 24 h of exposure, a study employed an in vitro intestinal co-culture model of Caco-2/HT29-MTX cells, treated with various concentrations (250–2500 mg/L) of both pristine and in vitro digested Ag NPs, as well as silver nitrate (Nanocomposix, US). A significant fraction of the Ag NPs dissolved (86–92% and 48–70%) during digestion. Cellular exposure to increasing concentrations of pristine or in vitro Ag NP-simulated digestion resulted in a concentration-dependent increase in total Ag and Ag NP content in the cellular fractions, measured by ICP-MS. The cellular concentrations were significantly lower following exposure to in vitro-simulated digestion of Ag NPs compared to the pristine Ag NPs, and Ag NP transport measured as either total Ag or Ag NPs was limited (<0.1%). Also, no Ag NPs could be detected, using TEM-EDS, in samples from the BL compartments in any of the exposure groups. The authors conclude that the surface chemistry of Ag NPs and their digestion influences their dissolution properties and uptake/association (measured by CLSM) with the Caco-2/HT29-MTX co-culture [50,51]. The co-culture of Caco-2/THP-1 was used to mimic and compare the intestine in a healthy (i.e., stable) and inflamed state. Co-cultures were exposed to concentrations between 0.01 and 10 μg/mL silver NPs (AgNPs) or silver nitrate (AgNO_3_) (Plasma-Chem GmbH Berlin, Germany) for 24 h. At higher concentrations of Ag NPs, a significant decrease (*p* ≤ 0.003) in TEER values of 8–14% was measured in all cell culture models [52]. In contrast, other authors observed no effect on barrier integrity following exposure of the 20 and 200 nm Ag NPs and Ag+ in Caco-2/TC7 monocultures and Caco-2/HT29-MTX co-cultures. No significant effects on monolayer integrity/permeability were observed after exposure to Ag NPs [53]. Moreover, another study found that Ag NPs impacted barrier integrity in an in vitro Caco-2/HT29-MTX co-culture only at the highest concentration tested (100 μg/mL) and after 96 h of exposure. It was shown that the Caco-2/HT-29MTX barrier exhibited high cytotoxic resistance to Ag NPs exposure. Uptake was observed in Caco-2 monocultures using CLSM after exposure to 50 μg/mL Ag NPs and in Caco-2/HT29-MTX co-cultures after 96 h of exposure to 100 μg/mL Ag NPs. However, no translocation across the intestinal barrier was detected [52]. After 24 h exposure to AgNO_3_, Kämpfer and collaborators showed TEER values increased significantly (*p* ≤ 0.003) in all experimental conditions, especially in the inflamed state. Ag NPs reduced the release of monocyte chemoattractant protein-1 (MCP-1) in the healthy model. Exposure to Ag NPs and Ag+ caused a significant increase in cell death with features of both apoptotic and necrotic processes performed by lactate dehydrogenase (LDH) release assay in both healthy and inflamed models even though the applied concentrations were negative in undifferentiated Caco-2 cells [51]. Several in vitro studies have shown the absence of Ag NP cytotoxicity; in detail, no cytotoxicity up to 100 μg/mL in Caco-2 monocultures and Caco-2/HT29-MTX co-cultures following exposure to Ag NPs of 20 and 200 nm was observed [52]. In a study conducted by Vila and colleagues, citotoxicity was detected after 24 h exposure with Ag NPs (8 nm) in undifferentiated Caco-2 monocultures with a EC50 of12.23 μg/mL [54]. A study demonstrated the cytotoxic effects on Caco-2 monocultures and Caco-2/HT29 co-cultures following single and repeated daily exposures (0.1–12 μg/mL) to colloidal silver Mesosilver™ and AgC for 18 days. Regardless of the cellular model, the uptake and intracellular localization of silver, analyzed using CytoViva™ and Helium Ion Microscopy with Secondary Ion Mass Spectrometry (HIM-SIMS), as well as the subsequent translocation of silver, were similar for both colloidal silver products. Specifically, CLSM analysis clearly showed that a significant number of NPs remained on the apical (AP) side of the membrane, retained between the microvilli and the mucus matrix. In both cases, some NPs were observed actively crossing the barrier [55].

Uptake and translocation were also demonstrated in Caco-2 monolayers following exposure to 1 or 5 μg/mL Ag (nanoComposix, San Diego, CA, USA) in the AP compartment. Specifically, the authors showed that both internalization and translocation to the BL side were greater for smaller Ag NPs than for larger ones, as determined by ICP-MS [56]. In a different study, the authors compared the effects of Ag NPs exposure on intestinal epithelia using a Caco-2 monolayer and a full-thickness 3D tissue model of the human small intestine (EpiIntestinal™). The Caco-2 monolayer exhibited a cytotoxic response after 24 h of treatment with 1 μg/mL Ag NPs. In contrast, neither genotoxicity nor cytotoxicity were detected in 3D tissues following NP treatment. Hyperspectral imaging using the CytoViva^®^ HSI microscopy system, along with ENVI^TM^ software and TEM, confirmed the uptake of Ag NPs by cells in both culture models studied [40].

In two other studies, limited silver translocation was also observed, with both total Ag and Ag NPs (<0.1%) detected after exposure of cells to Ag NPs that had been subjected to an in vitro gastrointestinal digestion process [50,57].

The results from a different study regarding particle coating are particularly interesting. Poly (acrylic acid) (PAA), used as the coating material for Ag NPs, was found to influence NP uptake, while translocation was determined by the core material [57].

In general, most of the analyzed studies found no changes in intestinal barrier integrity following exposure to less than 100 μg/mL of Ag NPs. In contrast, the ability of NPs to be taken up by intestinal cells was demonstrated by CLSM in both Caco-2 monocultures and Caco-2/HT29-MTX co-cultures after exposure to less than 100 μg/mL Ag NPs [40,54,56].

All papers highlight that Ag NPs can translocate across the intestinal barrier, even when the particles are Ag-coated [40,56,58].

Due to its physical and chemical properties, mucus may trap dietary inorganic NPs, such as Ag NPs, potentially reducing their toxicity to the host or, conversely, leading to high local accumulation and increased toxicity [55].

#### 3.1.5. Aluminum

Aluminum (Al) is increasingly used in food, consumer products, and packaging. It is present in human food and undergoes transitions between dissolved and particulate species during the passage of the GIT. Al NPs may occur as metallic (Al^0^) and insoluble oxidic species (Al_2_O_3_) as well as soluble Al chloride (AlCl_3_). Their solubility in biological media varies; for instance, metallic Al^0^ NMs release more ions than Al_2_O_3_, while AlCl_3_ presents both dissolved and agglomerated particulate forms. In the study of Sieg and colleagues [59], all these three Al species (particle dimension between 10 and 100 nm) were applied for 24 h to different co-culture models (Caco-2 monoculture, Caco-2/HT29 co-culture, M-cell model Caco-2/Raji-B co-culture). Cellular uptake occurred predominantly in particulate form, whereas ionic Al was not taken up by the intestinal barrier, and no transcellular transport was observed. None of the Al species showed cytotoxic effects up to 200 µg Al/mL. In a more recent study, the above-mentioned research group investigated the uptake and transport of various Al salts (citrate, sulphate, lactate, and acetylacetonate) using a Caco-2 monolayer in a Transwell^®^ system. The salts exhibited differing dissolution behaviors in biological media and formed nanoscale particles, inversely correlated with their dissolved fractions. Overall cellular uptake was low (<1% of applied Al), except for Al acetylacetonate, which exceeded 2%. In terms of translocation, aluminum citrate and acetylacetonate showed higher transport across the cell layer, at approximately 5% and 6%, respectively. The enhanced uptake and transport of Al acetylacetonate may be attributed to its lipophilic nature [60].

#### 3.1.6. Cerium Dioxide

Cerium dioxide (CeO_2_) NPs have a wide useful range of biological applications as antioxidants. The uptake and translocation profile of this NM was investigated by Vila and collaborators on Caco-2 monolayer after 24 h of exposure to several doses of CeO_2_ (∼80 nm) ranging from 1 to 100 µg/mL [61]. No toxic effects of CeO_2_ NPs were observed, as well as no effects on the monolayer integrity/permeability reported by either TEER measurement or LY passage. Very low CeO_2_ NP internalization was reported using fluorescent detection by confocal microscopy, since particles remained mostly attached to the AP membrane of the Caco-2 cells. Despite this apparent lack of uptake, a low translocation of CeO_2_ NPs to the BL compartment was observed only by confocal microscopy analysis.

#### 3.1.7. Iron

Iron (Fe) is a trace element that is abundant in human nutrition and highly relevant for essential vital functions. Iron oxide (Fe_2_O_3_) NPs are extensively employed in the biomedical sector, as well as in food colorant E172, where the presence of NPs has often been demonstrated to a very high extent [62]. However, concerns remain over the safety of iron oxide NPs due to incomplete toxicological data.

Internalization and transport experiments of Fe_3_O_4_ NPs were performed on both Caco-2 monoculture and two co-culture models (Caco-2/HT29-MTX E12 and Caco-2/Raji B cells) on Transwell^®^ systems. Pristine e PAA-coated particles were used. Only Fe_3_O_4_ NPs (8.2 nm) were transported through the cell barrier, indicating that the core material, not the coating, is the decisive factor for particle translocation to the BL side. In the Caco-2 monoculture model, Fe_3_O_4_ NP uptake was concentration- and time-dependent over 24 h, while translocation showed saturation at the highest concentration tested (80 µg/mL). In contrast, no saturation was observed for PAA-coated Fe NPs. A fraction of Fe_3_O_4_ NPs was localized within intracellular vesicles as agglomerates, with negligible ion release. Similar uptake and translocation profiles were observed in co-culture models, with approximately 2% of the administered dose internalized and 0.5% translocated. Barrier integrity assays (TEER and FITC-dextran) confirmed the absence of paracellular transport, and no cytotoxicity was detected [57]. Fe_3_O_4_ NPs remained largely undissolved during gastrointestinal passage (<5% ion release), reaching the intestine in particulate form. Uptake by Caco-2 cells was minimal and primarily mediated by the divalent metal transporter-1 (DMT1) [63]. In another study, Fe NP uptake ranged from 1.5% to 7.5%, depending on the tested NPs’ form (rod-shaped, spherical, or E172). However, no translocation to the BL of the Transwell^®^ system was detected for any of the Fe species tested at 50 µg/mL over 24 h [64].

In the further study of Sieg and collaborators, E172 at the initial concentration of 0.33 mg Fe/mL was added to 16 mL of artificial saliva [62], corresponding to the maximum recommended daily intake of iron supplements (6 mg/day) [65]. No significant iron release was reported during the artificial digestion process. Digested materials showed strong interaction with differentiated Caco-2 cells, with 20–30% of the administered Fe detected in the cellular fraction. However, as reported by Voss and colleagues, transepithelial transport remained very low. Finally, no cytotoxicity was observed on Caco-2 cells at concentrations of up to 100 µg Fe/mL within 24 h [64].

Uptake and translocation of digested Fe_2_O_3_ NPs (50 nm) were also investigated on Caco-2/HT29/Raji B tri-culture barrier model after 2 and 4 h of exposure to emulsions containing 0.05 and 0.01% Fe_2_O_3,_ diluted 1:3 in Dulbecco’s Modified Eagle Medium (DMEM) with or without Fetal Bovine Serum (FBS) [66]. The presence of FBS in the dilution medium increased cellular association of Fe_2_O_3_ NPs, with 22% and 46% associated after 2 and 4 h, respectively, compared to 13% and 31% in its absence. However, translocation to the BL was slightly higher without FBS, reaching 0.55% at 2 h and 1.77% at 4 h, versus 0.18% and 0.77% in the presence of FBS.

In conclusion, iron oxide NPs did not dissolve during gastrointestinal transit. Overall uptake was low, though it varied depending on NPs form, as shown in the study by Voss et al. In contrast, Sieg and collaborators reported strong interactions with the Caco-2 monolayer. Despite these differences, both studies observed minimal transepithelial transport. In the tri-culture model, a higher proportion of cell-associated Fe_2_O_3_ was found, particularly in the presence of FBS, even after short exposure times (22% at 2 h and 46% at 4 h). Nonetheless, translocation to the BL remained low [66].

#### 3.1.8. Copper

Copper (Cu) is an essential micronutrient present in all tissues and is required for many cellular functions. Copper oxide (CuO) NPs have antimicrobial properties and are used in many consumer products, such as textiles, food contact materials, and wood preservatives, due to their antifungal properties. CuO NPs are generally soluble and may, therefore, elicit toxicity via particle- and/or ion-mediated effects. For this reason, ionic controls (metal salt as copper sulphate—CuSO_4_) are often included in hazard studies. The effects of CuO NPs on Caco-2 monoculture and Caco-2/HT29-MTX and Caco-2/Raji B co-culture models were evaluated by Ude et al. in two different studies [67,68]. In the first study, 24 h of exposure to Cu at 6.34 and 12.68 μg/cm^2^ compromised intestinal barrier integrity, as evidenced by a reduction in TEER and ZO-1 staining, further supported by SEM analysis. The cellular uptake of CuO NPs (15–20 nm) was less than 3% of the initial exposure dose for all the concentrations tested, while translocation through the monolayer was concentration- and time-dependent. No difference was observed in the transport and uptake results between CuO NMs and CuSO_4_, suggesting that the effect of CuO NPs is partly mediated by ions [67]. The other study evaluated the impact of CuO NPs (15–20 nm) and CuSO_4_ on barrier integrity and translocation in Caco-2/Raji B co-culture and Caco-2/HT29-MTX co-culture. In both models, impairment of barrier integrity was observed through reduction in TEER values and immune staining of ZO-1. The reduction was more pronounced in the Caco-2/Raji B co-culture compared to the Caco-2/HT29-MTX co-culture, possibly due to the presence of mucus in the latter, which may limit nanoparticle–cell interactions. Additionally, ZO-1 staining reduction was less marked in the Caco-2/Raji B co-culture than in the Caco-2 monoculture, indicating reduced barrier disruption. These findings suggest that co-culture models are less sensitive to the effects of CuO NPs than the monoculture model [68]. Cu translocation to the BL was higher in the Caco-2/Raji B co-culture compared to the Caco-2/HT29-MTX model. In contrast, cellular Cu uptake was greater in the HT29-MTX co-culture, likely due to the retention of CuO NPs or Cu ions within the mucus layer, which remains adhered to the cell surface even after washing.

Results from the analyzed papers showed that after 24 h of exposure, CuO NPs induce barrier integrity impairment, cellular uptake, and translocation through the monolayer concentration- and time-dependent both on the Caco-2 monoculture model and on Caco-2/Raji B and Caco-2/HT29-MTX co-culture models. Co-culture models are less sensitive to Cu NPs effects than the Caco-2 monoculture model, while the presence of mucus in Caco-2/HT29-MTX co-culture appears to limit CuO NPs interactions with the cells and their translocation in the BL compartment but seems to increase the cellular uptake. CuO NPs effects are partly mediated by ions.

#### 3.1.9. Gold

Gold (Au) NPs are highly attractive to biomedical applications due to their size- and shape-dependent optoelectronic and physicochemical properties. Due to their desirable physicochemical properties, Au NPs hold great promise in industrial and advanced medical applications [69,70]. In one of the analyzed studies, Caco-2 monocultures grown on Transwell^®^ inserts were exposed apically and basolaterally to 5 µg/mL citrate–ligand-capped Au NPs of 10, 50, and 90 nm for 4, 8, and 24 h. NP characterization was performed by DLS, and uptake and transcellular transport were assessed using ICP-MS. Interestingly, BL exposure resulted in higher internalization than AP exposure, and larger Au NPs showed greater cellular uptake than smaller ones, regardless of the exposure side. Moreover, all tested Au NP sizes were detected on the BL side, indicating translocation across the cell layer [56]. In agreement with the previously mentioned paper, in another study, 10 min, 30 min, 1 h, and 4 h exposure of the Caco-2/HT29-MTX/THP-1 triculture model to 1 µg/mL Au NPs of different sizes and shapes (spheres: 30 nm and 200 nm; rods: 40 nm diameter × 112 nm length), characterized by TEM, resulted in a time-dependent increase in NP translocation to the Bl evaluated by ICP-MS [71]. Other authors analyzed the effects of 24 h exposure to 60 µM Au NPs on Caco-2 monocultures grown on Transwell^®^ inserts. Four Au NP types were tested, as follows: 15 nm spherical particles capped with either citrate or 11-mercaptoundecanoic acid (MUA), 60 nm spherical citrate-capped particles, and 60 nm MUA-capped star-shaped particles. All NPs were characterized by DLS prior to exposure. While both spherical and star-shaped 60 nm Au NPs were internalized by the cells, none of the tested NPs were able to cross the intestinal barrier, evaluated by graphite furnace atomic absorption spectrometry (GFAAS) method [72].

In conclusion, the existing literature indicates that Au NPs do not exhibit cytotoxicity; however, evidence of their internalization and translocation has been reported. Despite this, current data remain insufficient to allow definitive conclusions regarding their potential effects on the intestinal barrier.

### 3.2. Micro- and Nanoplastics

MNPs, mainly derived from the degradation of plastic waste, are solid polymer particles typically smaller than 5 mm and 100 nm in size, respectively [73]. MNPs are ubiquitous in the environment, contaminating water, air, and soil and becoming part of the food chain. Although there is limited information on the potential toxic effects and related mechanisms of action of these particles, they could involve oxidative stress, inflammatory reactions, DNA damage, and metabolism disorders [74]. Most of the analyzed literature data concern the effects of different types of polystyrene (PS MNPs), varying in size, shape, functionalization, and preparation. Among the other MNPs tested, there are polyethylene (PE), polymethylmethacrylate (PMMA), melamine formaldehyde resin (MF), polyvinyl chloride (PVC), and polymethacrylate (PMA) and biodegradable plastics, such as polylactic acid (PLA). One study investigating the effects of 50 nm uncharged polystyrene nanoplastics (PS NPs), characterized by TEM and DLS, reported no disruption of the intestinal barrier in a Caco-2/HT29-MTX co-culture model after 24 h exposure to concentrations ranging from 0 to 100 μg/mL, as assessed by LY permeability and TEER measurements [75]. In the study by Busch and collaborators, 24 h exposure to 0–50 μg/cm^2^ of positively charged, amine-functionalized 50 nm polystyrene NPs (PS-NH_2_), characterized by SEM and DLS, led to increased TEER and reduced ZO-1 expression in the Caco-2/HT29-MTX co-culture model. The authors attributed the elevated TEER to low-level necrosis, causing cell swelling and reduced intercellular spaces. The study proposes the use of PS-NH_2_ as a polymer-based particulate positive control when evaluating the toxicity of environmentally relevant polymeric particles [76]. As for negatively charged particles functionalized with surface carboxylic groups (PS-COOH), Hesler and collaborators reported no effect of 10 and 100 μg/mL 50 nm PS-COOH on barrier integrity (TEER) and translocation detected by Asymmetric Flow Field-Flow Fractionation (AF4) on the Caco-2/HT29-MTX co-culture after 24 h of exposure [77]. Another study investigated the translocation of PS NPs using fluorescence measurements via a Synergy HT Multi-Detection Microplate Reader, comparing particles of different surface charges (uncharged, -NH_2_, -COOH) and sizes (50 and 100 nm). After 24 h of exposure, uncharged 50 nm PS NPs and 50 nm PS-COOH showed the highest translocation across all in vitro intestinal models examined, including Caco-2 monocultures, Caco-2/HT29-MTX co-cultures, and Caco-2/HT29-MTX/Raji B tri-cultures [78]. In agreement with these observations, other studies have reported effects of uncharged PS NPs and PS-COOH on the intestinal barrier integrity and translocation rate [79,80,81,82]. In one of the aforementioned studies, the authors exposed various in vitro intestinal barrier models (Caco-2 monoculture, Caco-2/HT29-MTX co-culture, and Caco-2/Raji B M-cell model) to fluorescently labelled PS NPs of different sizes (20, 40, 100, 1000, 10,000 nm) and surface functionalizations (-NH_2_, -COOH) for 24 h, across a dose range of 1 × 10^10^ to 5 × 10^11^ µm^2^ particle surface/mL. Transport, assessed via fluorescence using a multi-well plate reader, was dependent on both particle size and surface chemistry. Positively charged particles showed enhanced cellular uptake and BL translocation [81]. The correlation between cellular uptake and/or transport and a positive charge agrees with previous studies [76,83]. A reduction in TEER values and a marked elevation of the LY concentration in BL of the Caco-2/HT29-MTX co-culture exposed for 24–48 h to 0–2000 μg/mL 20 nm PS-COOH were reported by Cui and co-authors [80]. In another study, the translocation (NanoDrop 3300 Fluorospectrometer) and barrier integrity (TEER) effects of uncharged PS NPs (50, 100, and 500 nm) were compared across intestinal barrier models (Caco-2 monoculture, Caco-2/HT29-MTX co-culture, and Caco-2/HT29-MTX/Raji B triculture) after 72 h exposure at 0.1 mg/mL. The presence of HT29-MTX cells reduced the translocation of 50 and 100 nm particles, likely due to mucus production, while the tri-culture model showed the highest translocation, possibly reflecting M-cell-mediated transport [79].

Several papers demonstrated that larger PS NPs—500, 1000, 4000, 5000, 10,000—uncharged or negatively charged, did not determine any adverse effect on intestinal barrier and were too large to be taken up by cells [77,81,82,84].

In the previously mentioned study, the Caco-2/HT29-MTX/THP-1 triculture model was exposed for 24 h to 0–50 μg/cm^2^ of PVC particles (~200 nm, characterized by SEM), and no adverse effects on barrier integrity, as measured by TEER, were observed [76]. In a separate study, the above-mentioned authors assessed the effects of PE MP particles with diameters ranging from 200 to 9900 nm (SEM data indicated most particles were below 1000 nm), again reporting no impairment of barrier function (TEER) in the same triculture model [85]. Moreover, two studies investigated the effects of a biodegradable plastic, the PLA microplastic, on Caco-2 monoculture model on inserts and Caco-2/HT29-MTX co-culture [86,87]. In one of the analyzed paper, TEM and SEM revealed the primary PLA particle size to be around 160 nm. In the Caco-2/HT29-MTX co-culture model, 24 h exposure to 0–100 µg/mL PLA resulted in particle internalization, confirmed by flow cytometry. TEER measurements indicated a slight but significant reduction in barrier integrity after 3 h, which was not observed at 24 or 48 h, possibly due to epithelial renewal repairing the initial damage [86]. Another study investigated the effects of PLA (250 and 2000 nm), PMMA (25 nm), and melamine formaldehyde resin particles (MF366, 366 nm) on different intestinal models, Caco-2 monoculture, Caco-2/HT29-MTX co-culture, and Caco-2/Raji B M-cell model. After 24 h exposure to 2.5 × 10^9^ µm^2^ particle surface/mL, no impairment of barrier integrity was observed in any model, as assessed by TEER and LY assay. Confocal microscopy revealed cellular uptake of all particles except 25 nm PMMA and fluorescence in the Bl indicated translocation of all tested particles [87].

Recently, several studies investigated the effects of MNPs following in vitro-simulated digestion. In a study employing the Caco-2/HT29-MTX/Raji B triculture model, 48 h exposure to 1, 5, and 20 µg/mL digested PS-COOH particles (25 nm and 1000 nm) did not elicit any disruption of barrier integrity, as indicated by TEER and FITC-dextran assays [88]. Another study compared the toxicity of different sized uncharged and negatively charged PS-MNPs 25, 100, 1000 nm subjected to in vitro-simulated digestion, till an effective oral concentration of 100 and 250 μg/mL. Using the Caco-2/HT29-MTX/Raji B triculture model, 24 h exposure revealed that, except for incinerated PE MNP particles (PE-I) and unmodified 25 nm PS NPs, all materials induced at least a two-fold increase in FITC-dextran paracellular permeability. A size-dependent uptake and translocation of PS-COOH was also observed, with the highest translocation for 25 nm and the lowest for 1000 nm particles [89]. These findings are consistent with those of Jabor and collaborators, who tested 143 μg/mL PS-COOH (43 ± 9 nm) and PS-NH_2_ (53 ± 15 nm) on Caco-2/HT29-MTX co-cultures for 72 h after in vitro-simulated digestion. All PS NPs were characterized by DLS in water and chyme, revealing increased particle size in chyme, likely due to agglomeration. TEER measurements showed a significant decrease following exposure to digested PS-NH_2_, while PS-COOH digests had no effect. Additionally, PMA-COOH (35 ± 6 nm) and PMA-NH_2_ (39 ± 9 nm) were tested at the same concentration, with PMA-NH_2_ inducing increased LY permeability and translocation, whereas PMA-COOH showed minimal translocation [90]. Another study evaluated the effects of 24 h exposure to digested PLA2000, PLA250, MF366, and PMMA25 (5 × 10^8^ µm^2^ particle surface/mL) on differentiated Caco-2 monocultures. Uptake and transport were compared to undigested particles. Results showed a reduction in cellular uptake and interaction for digested particles, likely due to strong agglomeration with intestinal organic matter, as evidenced by SEM. TEER and FITC-dextran assays indicated no barrier impairment [91]. Furthermore, in two studies of the same authors, the effects of PE-I 528.7 ± 16.4 nm were investigated using Caco-2/HT-29/Raji-B triculture model following in vitro-simulated digestion. After 24 h of exposure to 8.33 μg/mL PE MNPs, no significant impact on intestinal barrier permeability was observed, as demonstrated by the FITC-dextran assay [92,93].

Most studies concluded that MNP uptake, translocation, and barrier disruption depend strongly on particle size, shape, exposure time, concentration, and surface functionalization, with positively charged enhancing uptake and translocation, whereas negatively charged or uncharged particles exhibited lower or size-dependent activity. Moreover, the highest translocation of MNPs is often observed in tri-culture (Caco-2/HT29-MTX/Raji B) due to the presence of M cells [76,78,81,94,95]. MNPs <100 nm are more likely to cross the barrier; particles ≥500 nm show minimal or no uptake [77,81,82,84] In recent years, there has been growing scientific interest in the biological effects of biodegradable MNPs on human health. Among the analyzed studies, PLA was investigated in two works, both reporting no impairment of epithelial barrier integrity after 24 h of exposure, despite evidence of cellular interaction or internalization [86,87]. This could be either ascribed to the size or to the biodegradable nature of the particles.

Finally, the exposure to PS NPs, subjected to in vitro-simulated digestion, resulted in lower toxicity in the analyzed in vitro models compared to their pristine forms [88,90,91,92,93] and little translocation rate [90,91].

### 3.3. Nanocelluloses

Nanofibers encompass various types of dietary fibers produced at the nanoscale, such as nanocellulose and nanochitin, as well as organic or inorganic fibers of any other nature [6]. The most abundant organic polymer is cellulose primarily found in plant cell walls and in some algae, bacteria, and oomycetes. It is constituted by a polysaccharide made up of D-glucose units linked by β (1→4) glycosidic bonds whose molecular weight ranges from 50,000 to 2,500,000 depending on its origin and degree of polymerization.

Modified celluloses, like microcrystalline cellulose (MCC), are used in food applications due to their altered properties, which are achieved through processes like partial depolymerization. Cellulose, particularly in its nanostructured forms, is gaining interest for its wide range of applications, but it also requires careful assessment to ensure its safety, especially in food-related uses. NC is an emerging material with potential applications in various commercial fields, including medicine, energy, and food sectors, where it is used in food packaging (e.g., coatings and fillers) [96] or as food additives [97] or in novel foods.

There are two main types of NC, bacterial nanocellulose (BNC), produced by bacteria, and nanofibrillated cellulose (NFC) or cellulose nanocrystals (CNC), which are derived from plants or other materials.

NC materials exhibit a high aspect ratio, with different morphologies depending on the type (e.g., rod-shaped crystals for CNC and fibrils for NFC).

Certain dietary fibers are resistant to digestive enzymes in the human upper GIT, meaning that when they are nanosized, they may reach the small intestine while maintaining their nanoscale characteristics. As a result, it is essential to carefully examine the potential uptake and local effects of nanofibers in the small intestine to identify any potential risks. However, the diverse physicochemical properties of nanofibers may result in variations in local effects, gastrointestinal absorption, and any subsequent impacts.

However, the rising use of NC in food raises safety concerns, especially regarding its toxicokinetic behavior—whether nanosized particles can cross the intestinal epithelium and their potential selective uptake based on physicochemical properties.

The biological sources, production methods, and potential surface modifications of NC influence its physicochemical properties, such as size, aspect ratio, morphology, surface charge, and crystallinity. These factors lead to significant variations in NC characteristics both between and within types. This variability is crucial for the development of a growing number of applications [97,98].

Research for the hazard assessment of NC using NAMs was carried out in the EFSA-funded NANOCELLUP project. The study showed that the three different NC (BNC, CNC, and CNF) materials tested were internalized by the tri-culture intestinal barrier model. Additionally, immunotoxic effects were observed, including cytokine release and significant uptake of NC by macrophages, as well as the impairment of barrier function [99].

The hazard assessment of NC involves several complicating factors, such as the diversity of their physical characteristics, lack of standard reference materials, and analytical difficulties in their measurement [97]. Two animal studies showed no toxicity at high doses for two specific types of NC, but they may not have considered some early or specific toxic effects of NPs. Some in vitro studies found no cytotoxicity, but in an in vivo study, effects on oxidative stress and inflammatory response were observed after CNF treatment [100].

The impact on intestinal barrier function and cytotoxicity was studied in fully differentiated Caco-2 cells after 24 h of exposure to four types of nanocellulose crystals (CNCs) and four types of nanocellulose fibrils (CNFs) at a concentration of 50 μg/mL. There were four cellulose nanocrystals (CNCs)—CNC-140 nm × 20 nm; CNC-250 nm × 25 nm; CNC-700 nm × 25 nm; phosphoric acid cellulose nanocrystals (PCNC)-540 nm × 35 nm—and four cellulose nanofibrils (CNFs)—CNF-50 nm; CNF-80 nm; CNF-30 μm; TEMPO (2,2,6,6-tetramethylpiperidine-1-oxyl radical) oxidized CNF (TCNF)-250 nm × 25 nm. The effect of 24 h exposure to eight different CNMs on the integrity of the Caco-2 cell monolayer was assessed using 3000 Da AF488-dextran and immunofluorescence staining of TJs. While none of the CNMs significantly altered the permeability (Papp) for AF488-dextran, there was a slight increase in permeability following exposure to the CNFs. Immunofluorescence results supported the permeability findings, showing no disruption of the TJ band between cells. However, while the four CNCs did not affect the localization of the TJ protein ZO-1, aggregates of ZO-1 were observed in the cytosol for CNF-50 nm and, to a lesser extent, for CNF-80 nm suggesting that these two CNFs may affect TJ organization without compromising TJ integrity. Two CNMs (CNC-250 nm × 25 nm and CNF-30 μm) were found to cause a slight but significant increase in cell membrane permeability, suggesting that these two materials adversely impact the enterocyte cell surface. Of the eight tested CNMs, three significantly decreased mitochondrial activity (CNC-250 nm × 25 nm, CNF-80 nm, and CNF-30 μm) [101].

Recent study results show that CNC crosses the intestinal mucus layer but does not pass the intestinal tissue barrier in in vitro intestinal triculture model (Caco2/HT29-MTX/THP-1) following 2 h exposure to 100 μg/mL; furthermore, no toxic effects were observed under exposure conditions tested [102].

In the EFSA-funded NANOCELLUP project, cellular uptake was demonstrated in an in vitro intestinal triculture model after repeated exposure to 30 µg/mL concentration of CNC [99].

So far, data regarding uptake and crossing are conflicting; in one study, fluorescently labeled CNC was used to test whether the NC could cross a monolayer of Caco-2 cells with an additional mucus layer. The results suggested that upon treatment with CNC in 0.5% (*w/w*), the nanofibers were unable to penetrate the Caco-2 cell monolayer with a mucus layer [98]. The papers analyzed highlight that the diameter and length of CNMs play an important role in the interaction between these materials and the enterocyte monolayer in vitro.

Furthermore, NC can impact on uptake of nutrients, as shown by a study in which there was an increased uptake of glucose from the digesta of starch solutions in the presence of NC, measured in Caco-2/HT29-MTX/Raji B tri-culture model [103]. Nutrient uptake in the GIT is normally not included in toxicity studies but needs further consideration to enable assessment of their impact in the long-term studies.

## 4. Discussion

The results collected in this scoping review highlight how the NM uptake and translocation across the in vitro intestinal barrier are strongly modulated by a combination of physicochemical properties and biological factors. Key parameters such as size, shape, surface charge, and functionalization or coating critically affect their interaction with the intestinal epithelium. Smaller NMs (<100 nm) tend to cross the barrier more efficiently via endocytic pathways, while positively charged particles may exhibit stronger adhesion to the negatively charged cell membranes, promoting uptake. Surface modifications can further modulate NM biointeractions, reducing aggregation, and impacting on translocation efficiency. The mucus layer, composed mainly of mucins forming a gel-like network, serves as a steric and electrostatic barrier to NPs diffusion. Its physical properties, such as thickness and viscosity, can significantly influence NM behavior [7].

Barrier integrity, monitored through TEER measurements or passage of specific markers, such as LY or FITC-Dextran, modulates paracellular transport, and its disruption may facilitate increased translocation of NMs. Finally, experimental parameters, such as exposure time, NM concentrations, and NM dispersion protocols, significantly influence the reliability and interpretation of uptake and crossing data.

**Figure 2:**
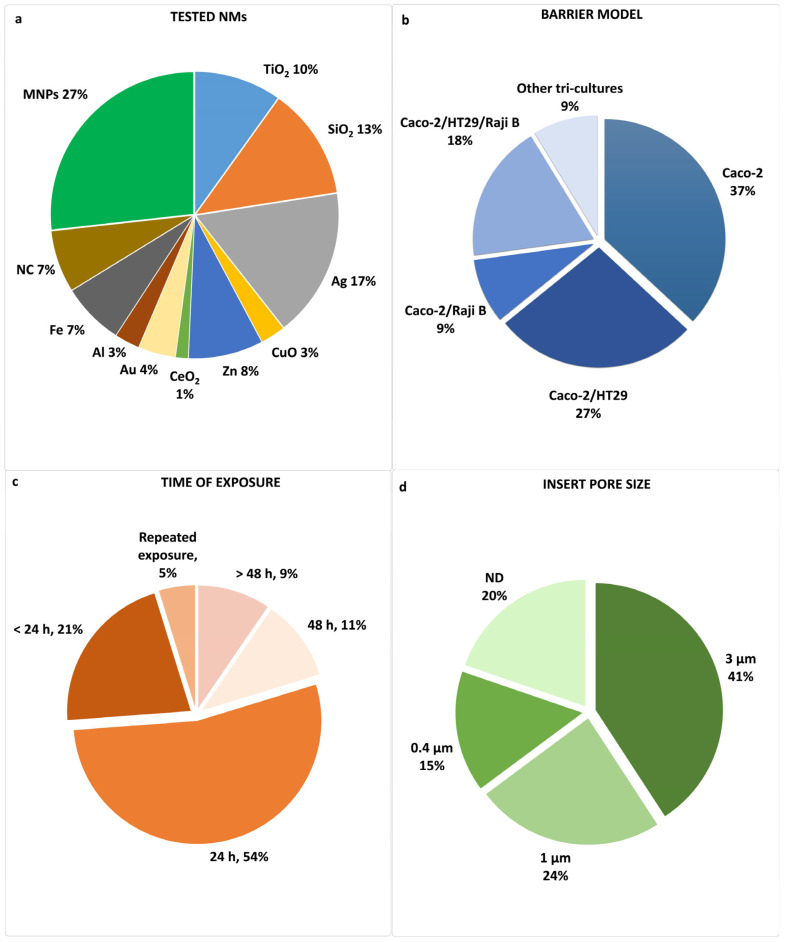
Analysis of each NMs (%) of the total paper considered (**a**); the types of barrier models (**b**); the time of exposure (**c**); and insert pore sizes (**d**) of the intestinal barrier models included in the studies of the present review.

The types of NMs investigated and the key features of the in vitro barrier models employed, expressed as percent ages relative to the total number of studies included in this review, are summarized in Figure 2.

Specifically, Figure 2a shows the different NMs studied, Figure 2b the main types of in vitro barrier models utilized, Figure 2c the most frequent testing exposure times, and Figure 2d the insert pore size used for the intestinal barrier models.

Among the inorganic materials, the most studied are Ag (17%), SiO_2_ (13%), TiO_2_ (10%), Zn (8%), and Fe (7%). Whereas, the most investigated organic NMs are MNPs (27%) and NC (7%) (Figure 2a).

As described in Figure 2b, the most used intestinal in vitro barrier model for accumulation and crossing studies is represented by Caco-2 monoculture (37%) followed by the Caco-2/HT-29 MTX co-culture model (27%) and the more complex triple Caco-2/HT-29 MTX/Raji B tri-culture model, able to simulate the M-cell phenotype (18%).

Regarding exposure conditions, approximately 50% of the reviewed studies employed a 24 h exposure duration (Figure 2c), despite the Caco-2 model being suitable for longer or repeated exposures. Most investigations focused on short-term treatments, which may underestimate the potential toxic effects of NMs under more realistic, chronic exposure scenarios. Assessing extended exposure durations is therefore essential for a more accurate evaluation of cumulative risks [53].

The pore size of the inserts (Figure 2d) is also a critical parameter, particularly in translocation studies, as it can significantly influence NP passage across the membrane. Studies indicate a preference for larger pore sizes (3 µm, used in 36% of cases) over smaller ones (1 µm and 0.4 µm).

**Figure 3:**
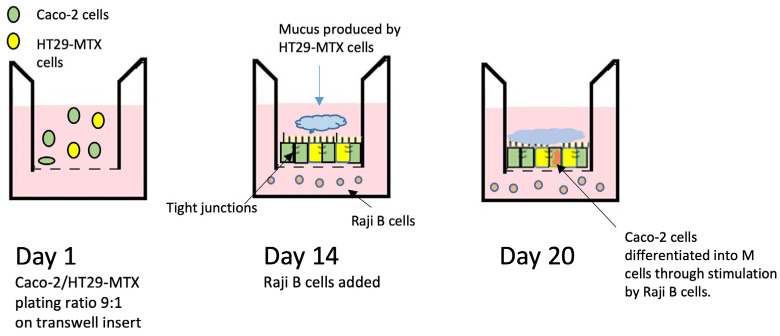
Schematic representation of the Caco-2/HT29-MTX/Raji-B in vitro intestinal barrier model set-up.

Based on the reviewed papers, the Caco-2/HT29-MTX/Raji B triculture model (Figure 3) has emerged as one of the most physiological in vitro systems for mimicking the human intestinal barrier. Multiple studies have demonstrated that this model closely reproduces both the structural complexity and functional properties of the intestinal epithelium, thereby representing a physiologically relevant in vitro system for toxicological assessments. HT29-MTX, mucus-producing cells, are a critical component in in vitro intestinal barrier models designed to evaluate NMs uptake and translocation. These cells secrete a mucus layer that closely mimics the native intestinal environment. Moreover, Raji B cells are essential for promoting the induction of Caco-2 cells into M-like cells, which play a key role in particle translocation across the intestinal epithelium, thereby enhancing the relevance of the model for studying the transport of NMs. M cells, located in the follicle-associated epithelium of Peyer’s patches, are specialized in uptake and transcytosis of macromolecules and microorganisms from the gut lumen to immune cells across the epithelial barrier. Although they represent only ~1% of intestinal epithelial cells and remain poorly characterized, their unique morphology—lacking apical microvilli and featuring a basolateral “pocket”—supports their role in antigen sampling and immune cell interaction.

While Caco2-Raji B co-culture systems are commonly employed to simulate M cell-mediated NP uptake and possible translocation, only a limited subset of the studies included in this review (6 out of 24 that report M cell presence in the co-culture system) provided direct validation of M-cell differentiation, through ultrastructural analysis. The lack of such confirmation in several cases may introduce uncertainties in data interpretation and suggests that further validation efforts could strengthen the reliability and biological relevance of this model in NP transport studies.

Concerning the characterization of the (co)-culture models, the following three parameters are generally considered: (i) barrier integrity measured by TEER and/or passive passage of fluorescent markers as LY or FITC-dextran, (ii) mucus production usually detected by Alcian blue staining, and (iii) M-cell identification, the less standardized parameter, mainly determined by wheat germ agglutinin (WGA) assay or by TEM imaging.

### 4.1. Overview of the NMS Effects on Intestinal Barrier Models

For inorganic NPs, most of the studies reported the absence of barrier impairment after exposure to TiO_2_, ZnO, SiO_2_, Al, CeO_2_, Fe, Cu, and Au NPs [24,38,39,40,43,56,61,104,105]. Conversely, some studies reported size-, concentration-, and time-dependent toxicity as well as barrier impairment for Ag, TiO_2_, Cu, and SiO_2_ NPs [23,41,42,51,53,67,68]. Moreover, all the above-mentioned NPs were observed to be internalized by intestinal cells [7,23,38,40,41,42,44,56,61,66,68,71,72].

As previously discussed, the presence of mucus in Caco-2/HT29-MTX co-culture models acts as a barrier to NPs uptake and translocation. Particularly, mucus effect has been reported for ZnO, SiO_2_, for which the accumulation in the mucus is correlated with particle size increase, and for CuO [28,41,42,68].

Low translocation rate through intestinal epithelium is generally observed for some of the inorganic NPs considered in this review as TiO_2_, ZnO, Ag, Al, CeO, Fe, and Au [24,28,54,56,60,61,62,64,66,71].

Concerning MNPs, most of the analyzed studies concluded that uptake, translocation, and barrier impairment of MNPs were strongly affected by size, time of exposure, concentrations, and functionalization, with positively charged (-NH2) PS NPs noticeably leading to an increased cellular uptake and transport compared to negatively charged (-COOH) or uncharged PS NPs [76,78,81,95]. In addition, the presence of HT29-MTX cells inhibited the translocation, likely due to mucus production, and the highest translocation occurred in the tri-culture model, possibly attributed to the presence of M cells [79].

Data regarding nanofibers’ uptake and crossing are conflicting; some studies reported that nanofibers were unable to penetrate the Caco-2 cell monolayer with a mucus layer [99]. In contrast, other authors suggest that NC could impact the uptake of nutrients. Nutrient uptake in the GIT is normally not included in toxicity studies but needs further consideration to enable assessment of the impact in the long-term studies [104].

In general, the highest translocation rate was detected in the Caco-2/Raji-B co-culture followed by the Caco-2/HT29-MTX/Raji-B tri-culture model, suggesting that M-cells act as portals for NP cell translocation, and the mucus layer modulates their passage [7,78]. However, material-specific variations exist, highlighting the need to consider both NP characteristics and barrier complexity in evaluating intestinal translocation.

In conclusion, both inorganic and organic NMs can interact with the intestinal barrier, but their impact varies widely depending on particle type and properties; in accordance with the nanotoxicology paradigm, smaller particle sizes are often associated with increased potential toxicity. Currently, some NPs are considered safe, while others raise safety concerns, highlighting the importance of particle-specific hazard assessments and further targeted investigations, particularly under long-term and realistic exposure conditions.

### 4.2. Data Reuse

The ability to reuse the available experimental data produced in different contexts (e.g., in the research or regulatory environment) is of the utmost importance. Data producers should provide enough detail to allow others to understand the experimental design, interpret the results accurately, and reuse the data effectively. However, many publications describing in vitro experiments lacking essential information, making it difficult to replicate experiments or assess result reliability. This limits their scientific value and can lead to the unnecessary duplication of research.

To facilitate data reuse, the adoption of standardized reporting formats, such as the OECD Harmonized Templates (OHT), are essential. However, many NAMs currently lack dedicated OHTs, as they are not yet included in OECD guidelines. Some existing OHTs have been adapted for NMs, and this process is still ongoing. In response, several EU nanosafety initiatives are developing reporting templates to ensure consistent data capture and enable integration into databases, such as eNanoMapper (https://enanomapper.adma.ai/, accessed on 12 June 2025). These platforms, developed within the EU FP7 eNanoMapper project, form part of the computational infrastructure for managing toxicological data on engineered NMs and are aligned with the FAIR principles (Findable, Accessible, Interoperable, and Reusable), which promote both human and machine accessibility to data [106,107].

Among initiatives within international regulatory authorities and agencies, it is worth highlighting EFSA, which has emphasized the importance of applying FAIR principles to both data and methods within its qualification framework for NAMs [108]. Integrating NAMs into a FAIR-aligned framework aims to improve transparency, reliability, and regulatory acceptance of these alternative methods. This is particularly important in the nanosafety field, where the complexity and heterogeneity of NM data pose major challenges. To address these needs, EFSA has proposed a FAIR-based qualification system for NAMs, to standardize the description, sharing, and reuse of data and methods, thereby improving reproducibility and supporting more robust safety assessments.

The reviewed literature clearly highlights the critical need for a human in vitro intestinal barrier model to study NM uptake and translocation following oral exposure, with important implications for safety and regulatory assessment. When aligned with FAIR principles, such a model improves data quality, transparency, and reproducibility. FAIR-compliant data also facilitate regulatory acceptance, cross-study comparisons, and model reuse. As such, a well-designed, FAIR-compliant model is essential for advancing nanosafety research and supporting ethical, robust regulatory practices.

## 5. Conclusions

Intestinal uptake and translocation studies are essential for human NM safety assessment, as they provide indications about NMs’ entry into the body and their potential distribution to secondary target organs. In vitro intestinal models provide practical tools allowing the assessment of both cellular uptake and translocation supporting human oral exposure risk assessment. A large amount of data on NM nanosafety is already available, providing a valuable foundation for future research and regulatory advancements in NMs risk assessment. Emphasis is placed on the use of in vitro intestinal barrier models, incorporating enterocytes, M cells, and mucus-producing goblet cells, representing a promising NAM for assessing NM uptake, translocation, and barrier impairment under physiologically relevant conditions, with potential applications in regulatory safety assessment and hazard identification frameworks.

Nevertheless, the information collected in the literature for this scoping review has highlighted the presence of gaps in knowledge relevant to the development of the optimal intestinal barrier protocol for integrated approach purposes as well as for inter-laboratory comparison.

Main gaps identified are, as follows:

- Limited characterization and standardization of the co-culture models, particularly for which concern M cells identification
- Lack of comparison between mono-, bi-, and tri-culture models in respect to NM effects
- Some systematic studies on NM translocation and uptake
- Some data on NMs exposure longer than 24 h and on NMs’ repeated exposure
- Limited studies conducted applying dose range within the human daily intake

In conclusion, this scoping review highlights the growing robustness and relevance of the in vitro intestinal barrier model in the safety assessment of NMs relevant to oral exposure, as they address critical toxicokinetic endpoints. The advancement of these models from simple monocultures to more complex tri-culture systems has notably contributed to the generation of substantial high-quality data across various chemical categories of NMs. A FAIR-compliant in vitro intestinal model is crucial for investigating oral NM uptake, enhancing data quality and reproducibility, supporting regulatory decision making, and advancing nanosafety research. Despite existing gaps in methodology and standardization, these approaches are expected to remain valuable, particularly with improved interlaboratory reproducibility and increased regulatory acceptance.

Finally, future developments, such as intestinal organoids, intestinal reconstructed tissues, and fluidic and mechanical stimulated models, which better simulate the complexity of the intestinal architecture, are emerging as promising tools for nano risk assessment, offering a more physiologically relevant, human-derived platform to evaluate the safety and toxicological impacts of NMs.

## Figures and Tables

**Table 1:** Number of articles retrieved for each “search terms”.

Search Terms	PubMed	Scopus
Nanoparticle AND intestinal in vitro AND uptake/accumulation	425	491
Nanoparticle AND intestinal in vitro AND crossing/translocation	155	55
Nanoparticle AND intestinal in vitro AND barrier integrity	55	66
Titanium oxide nanoparticles AND intestinal in vitro AND uptake/accumulation OR crossing/translocation OR barrier integrity	20	9
Zinc oxide nanoparticles AND intestinal in vitro AND uptake/accumulation OR crossing/translocation OR barrier integrity	10	18
Silica oxide nanoparticles AND intestinal in vitro AND uptake/accumulation OR crossing/translocation OR barrier integrity	38	10
Silver nanoparticles AND intestinal in vitro AND uptake/accumulation OR crossing/translocation OR barrier integrity	37	45
Aluminum nanoparticles AND intestinal in vitro AND uptake/accumulation OR crossing/translocation OR barrier integrity	5	1
Cerium nanoparticles AND intestinal in vitro AND uptake/accumulation OR crossing/translocation OR barrier integrity	7	2
Copper nanoparticles AND intestinal in vitro AND uptake/accumulation OR crossing/translocation OR barrier integrity	4	0
Iron nanoparticles AND intestinal in vitro AND uptake/accumulation OR crossing/translocation OR barrier integrity	38	6
Gold nanoparticles AND intestinal in vitro AND uptake/accumulation OR crossing/translocation OR barrier integrity	27	6
Microplastic OR nanoplastic AND intestinal in vitro AND uptake/accumulation OR crossing/translocation OR barrier integrity	120	51
Nanocelluloses OR nanofibers AND intestinal in vitro AND uptake/accumulation OR crossing/translocation OR barrier integrity	14	1

**Table 2:** List of elements considered as inclusion/exclusion criteria.

	Inclusion Criteria	Exclusion Criteria
**Model Type**	Studies using human in vitro intestinal barrier models Source and description of biological models	Studies using not human in vitro models or acellular system
**Focus of Study**	Studies investigating barrier impairment, uptake, or translocation of nanoparticles across in vitro human intestinal barrier	General studies on cytotoxicity not mentioning uptake or crossing Studies reporting poor quality data
**Material Type**	Studies including source and description of the nanomaterial Studies using engineered nanoparticles, nanomaterials, nanocarriers, or nano-formulations intended for oral or gastrointestinal exposure	Studies focusing on non-nano materials
**Publication Type**	Original research articles with experimental data Peer review papers	Duplicates studies: the most recent one was included Non original research Non-peer-reviewed articles or grey literature Reviews, conference abstracts, editorials, letters, commentaries, or patents

**Table 3:** Parameters and endpoints analyzed within this review.

**Physical–chemical parameters**	NMs identification (size, shape, coating, material physical state, use of dispersants) NMs characterization Dispersion protocol
**Experimental parameters**	NMs dose Time of exposure Cell culture model Model characterization Insert pore size Detection techniques (ICP-MS ^1^, ICP-OES ^2^, others)
**Endpoints**	Barrier impairment NMs uptake NMs translocation

^1^ Inductively Coupled Plasma Mass Spectrometry. ^2^ Inductively Coupled Plasma Optical Emission Spectroscopy.

## Data Availability

Data used in this study are publicly available in the sources cited in the method and result sections.

## Supplementary Materials

The following supporting information can be downloaded at https://www.mdpi.com/article/10.3390/nano15151195/s1, Table S1: Detailed information on the number of papers retrieved through the search strategy, the number of papers excluded before screening, the number of papers excluded according to inclusion/exclusion criteria, and the final number of papers analyzed, for each search term used in the present review; Table S2: Key details of the studies conducted in the papers included in this review (e.g., author(s), year of publication, NM type and size, cellular model, treatment dose and exposure time, uptake/crossing result, and main findings).

## Abbreviations

The following abbreviations are used in this manuscript:

AP	Apical
BL	Basolateral
BNC	Bacterial Nanocellulose
CLSM	Fluorescence Confocal Microscopy
CNC	Cellulose Nanocrystals
DLS	Dynamic Light Scattering
DMEM	Dulbecco’s Modified Eagle Medium
EC	European Commission
EC50	Effective Concentration 50
EFSA	European Food Safety Authority
EMA	European Medicines Agency
EU	European Union
FAE	Follicle-Associated Epithelial
FAIR	Findable, Accessible, Interoperable, and Reusable
FBS	Fetal Bovine Serum
FDA	Food and Drug Administration
FITC-Dextran	Fluorescein Isothiocyanate-Labelled Dextran
GIT	Gastrointestinal Tract
HTS	High-Throughput Screening
IAP	Intestinal Alkaline Phosphatase
ICP-MS	Inductively Coupled Plasma Mass Spectrometry
ISO	International Organization for Standardization
LDH	Lactate Dehydrogenase
LY	Lucifer Yellow
M-cells	Microfold Cells
MCC	Microcrystalline Cellulose
MF	Melamine Formaldehyde Resin
MNPs	Micro-Nanoplastics
MUA	Mercaptoundecanoic Acid
NAMs	New Approach Methodologies
NC	Nanocellulose
NFC	Nanofibrillated Cellulose
NMs	Nanomaterials
NPs	Nanoparticles
OECD	Organisation for Economic Co-operation and Development
OHT	OECD Harmonized Templates
PAA	Poly Acrylic Acid
Papp	Apparent Permeability Coefficient
PE	Polyethylene
PLA	Polylactic Acid
PMA	Polymethacrylate
PMMA	Polymethylmethacrylate
PS	Polystyrene
PVC	Polyvinyl Chloride
SAS	Synthetic Amorphous Silica
SEM	Scanning Electron Microscopy
sp-ICP-MS	Single-Particle Inductively Coupled Plasma Mass Spectrometry
TEER	Transepithelial Electrical Resistance
TEM	Transmission Electron Microscopy
WGA	Wheat Germ Agglutinin
ZO-1	Zonula Occludens-1
